# Jejunal diverticulosis - A case series and literature review

**DOI:** 10.1016/j.amsu.2022.103477

**Published:** 2022-03-03

**Authors:** Douglas Chung

**Affiliations:** Campbelltown Hospital, Therry Road, Campbelltown, NSW, 2560, Australia

**Keywords:** Case report, Jejunal diseases, Diverticululum, Intestinal perforation

## Abstract

**Introduction:**

Scant literature is available regarding in vivo jejunal diverticulosis, in part due to its typically asymptomatic course. This is made more difficult by the difficulty in establishing its diagnosis. This case series examines a number of patients presenting to our hospital with jejunal diverticular disease, and their varying clinical courses.

**Methods:**

A number of cases that had presented to our hospital with jejunal diverticulosis were reviewed retrospectively in keeping with PROCESS guidelines. Their presentations, investigations, and management rationale are discussed in brief.

**Discussion:**

The presentation of jejunal diverticulosis is varies significantly along a spectrum, with a number of symptoms similar to other common intra-abdominal pathologies. The imaging modalities of choice are a barium small bowel series, CT scans, and enteroclysis, varying in sensitivity and complexity. Decision making with regards to operative vs. non-operative management is typically in line with that of colonic diverticulosis, though no strict guidelines have been established.

**Conclusion:**

Jejunal diverticulosis is an uncommon, with scarce data available on the appropriate investigation and management pathways. Its presentation is difficult to differentiate from other intra-abdominal pathology, and its investigations either poorly sensitive or costly and technically challenging. The general consensus on its management is similar to that of colonic diverticula, though more research needs is warranted.

## Highlights

• Jejunal diverticulosis is an uncommon cause for non-specific abdominal pain.
• It is infrequently suspected due to the difficulty in its diagnosis.
• Presentations range from pain, to bleeding, to an acute abdomen.
• Diagnostic modalities include barium small bowel series, enteroclysis, and CT scans.
• Management is similar to that of colonic diverticula.

## 1 Introduction

Jejunal diverticulosis is an elusive entity, with reported incidence rates of 0.5% [1], with clear documentation of its existence in both intraoperative and post-mortem examinations, but scant literature regarding in vivo disease. This is in part due to its largely asymptomatic course. Another significant barrier is difficulty in establishing its diagnosis [2,3], despite utilisation of various modalities in its assessment, such as barium small bowel series [3], CT scans [3], and enteroclysis [1]. This case series examines a number of patients presenting to our hospital with jejunal diverticular disease, and their varying clinical courses.

## 2 Methods

This study is a retrospective case series of individual cases presenting at a single tertiary hospital over the course of 6 months examining the spectrum of presentations associated with jejunal diverticulosis. The cases are presented in keeping with the PROCESS guidelines [10]. Their presentations, investigations, and management rationale are discussed. This study has been registered to the research registry with the UIN researchregistry7695.

## 3 Case 1

**Fig. 1:**
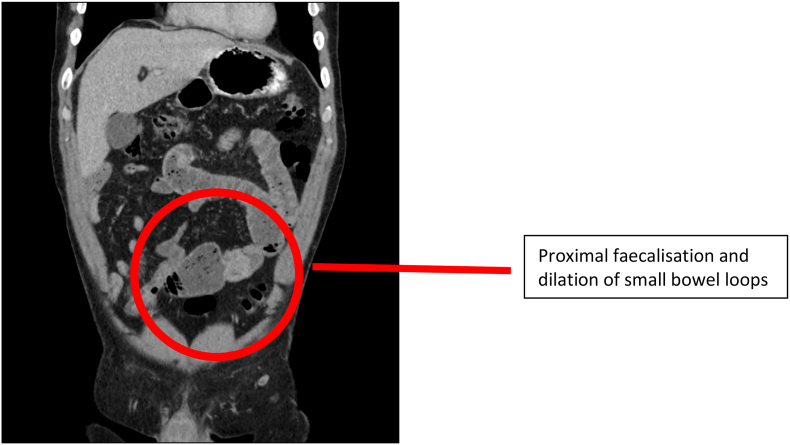
Coronal CT scan of intussusception.

**Fig. 2:**
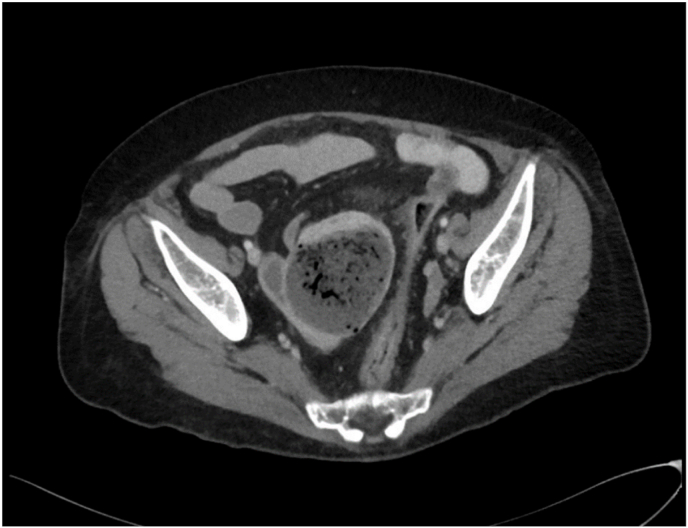
Pre-operative CT findings.

A 55 year old gentleman presented with abdominal pain, vomiting, and constipation (without obstipation). He was incidentally noted to have had dysphagia of 12 months, and had undergone a recent gastroscopy and colonoscopy, both of which did not identify any abnormalities. Her background included multiple lipoma excisions, but was otherwise unremarkable. He had markedly raised inflammatory markers. His CT scan identified small bowel intussusception [Fig. 1, Fig. 2]. Incidentally, he was found to have a desmoplastic lesion in his right iliac fossa, with irregular thickening of the terminal ileum, alongside multiple enlarged adjacent lymph nodes suggestive of a potential lymphoma or carcinoid tumour. He was tachycardic throughout his presentation.

A decision was made to undertake a diagnostic laparoscopy to assess the nature of his intussusception. Intraoperatively, a constriction was noted 30 cm from his duodenojejunal flexure on a small bowel run, and the procedure was converted to a laparotomy. Innumerable enlarged, firm lymph nodes were noted in a thickened mesentery. The intussuscepted segment was resected. The remnant bowel was anastamosed primarily. 4 jejunal diverticulae were noted incidentally during the remaining small bowel run, adjacent to each other, in the mid jejunal region, with each measuring approximately 1 cm in diameter. Given the nature of the ongoing procedure, they were handled with care, but otherwise left intact.

Post-operatively, the patient experienced a slow but uneventful recovery. His pathology returned as a T4 poorly differentiated adenocarcinoma.

## 4 Case 2

A 74 year old lady presented with pain in the right iliac fossa over the course of 1 week, with associated nausea and vomiting. Her background medical issues included hypothyroidism. She had raised inflammatory markers. Her examination revealed tenderness in the right iliac fossa without peritonism. A CT scan was performed to identify a cause for said pain, which identified acute jejunal diverticulitis with a localised perforation. This appeared as a 33 × 22mm inflammatory mass containing a small volume of gas, localised to the left iliac fossa. She was haemodynamically stable throughout her presentation, prompting a decision to undertake non-operative management of her diverticulitis.

In line with the local hospital policy, she was commenced on ceftriaxone and metronidazole. Her recovery was uneventful, and she was discharged home to follow up with gastroenterology to organise an outpatient gastroscopy and colonoscopy.

## 5 Case 3

A 69 year old lady presented with abdominal pain, vomiting, and low grade fevers of 1 day. Her background medical issues include ulcerative colitis, for which she was on a biological agent; chronic lymphocytic leukaemia; and hypertension. Her inflammatory markers were chronically raised, making interpretation difficult. Her examination revealed a peritonitic abdomen, prompting an urgent CT scan, which revealed a faeculent walled off collection measuring 56x58 × 60mm abutting the small bowel loops in her pelvis. She remained haemodynamically stable, albeit borderline hypotensive with IV fluid resuscitation.

**Fig. 3:**
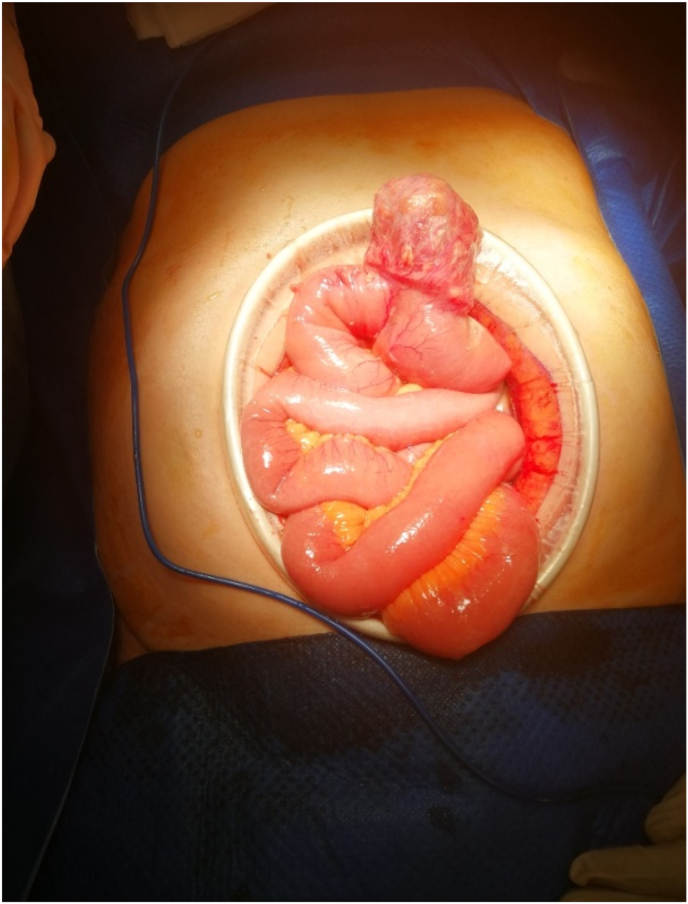
Intraoperative photo.

This clinical picture prompted an immediate transfer to theatres, during which the patient underwent a laparotomy. This revealed a contained perforation attached to the proximal jejunum on its anti-mesenteric border [Fig. 3]. Its contents were frankly faeculent, and a decision was made to resect a 12 cm section of small bowel including the perforation.

Her recovery was uneventful, and she was discharged home 1 week post-operatively.

## 6 Discussion

The above cases serve to illustrate the different ways in which jejunal diverticulosis present, from a benign incidental finding to a surgical emergency requiring prompt intervention. While non-exhaustive, it serves to demonstrate how spectrum and appropriate forms of management of the disease.

The vast majority of these cases remain asymptomatic [5] and are generally found incidentally, given the difficulty in its diagnosis. It is most frequently diagnosed on CT scanning, in which it is described as a ‘discrete round or ovoid, contrast-, fluid-, or air-containing structures outside the expected lumen of the bowel [3]. Less commonly, double balloon enteroscopy is utilised, with limited success [4]. The ideal modality for diagnosis of this entity appears to be enteroclysis [2], utilising methylcellulose to increase bowel distension, thereby improving the diagnostic yield of the contrast agent following it.

While not commonly symptomatic, jejunal diverticulosis can present with a variety of symptoms, the mildest of which include vague abdominal pain [2], distension, nausea [6], and bleeding per rectum [7]. This overlaps significantly with a plethora of intra-abdominal conditions, further complicating diagnosis. Less commonly, it may present as gram negative sepsis [8]. More complicated disease can present as a perforation [6,9], presenting with similar symptoms in a more severe fashion, typically necessitating emergency surgery.

A decision of operative vs. non-operative management is unclear at present. As most asymptomatic and minimally symptomatic cases documented resolve spontaneously with no sequelae, this author advises a conservative approach and treatment of complications as they arise. However, there have been suggestions [5] for more aggressive therapy with resection, given the increased likelihood of its perforation compared to other forms of non-Meckeliandiverticulosis. Perforations are almost universally resected [2,6,9], especially in patients who are haemodynamically unstable with signs of intra-abdominal sepsis and pneumoperitoneum. However, our trial of non-operative management for case 2 suggests this form of management may play a role in management algorithms. Strong evidence is lacking with regards to appropriate management of this disease, and further research is necessary in looking to create a universal management guideline.

Decision making becomes further complicated when the perforated portion of bowel is not identified during laparotomy, despite the presence of multiple diverticula. In these scenarios, limited evidence suggests washout and closure of the abdomen [5], to which wide drainage should be added as appropriate.

Ultimately, this disease is likely to present as an indolent longstanding abdominal pain. As such, future research should be undertaken in the outpatient setting, ideally in a prospective fashion for non-specific abdominal pain, though limitations in case number will necessitate initial utilisation of retrospective data.

## 7 Conclusion

Jejunal diverticulosis is an uncommon, with scarce data available on the appropriate investigation and management pathways. Its presentation is difficult to differentiate from other intra-abdominal pathology, with non-specific symptoms that could be mistaken for colonic diverticulitis, appendicitis or any number of common presentation. Its investigations are either poorly sensitive or costly and technically challenging, requiring a high index of suspicion before any investigations are undertaken. The general consensus on its management is similar to that of colonic diverticular disease, though more research needs to be done with regards to a pathway for its management. It is hoped that this paper is to increases awareness and consideration of this clinical entity as an alternative diagnosis in oft found non-specific abdominal pain.

## Ethical approval

Written informed consent was obtained from the patient for publication of this case report and accompanying images, in line with local ethical approval requirements. No other requirements were stipulated.

## Sources of funding

This research did not receive any specific grant from funding agencies in the public, commercial, ornot-for-profit sectors.

## Author contribution

Douglas Chung - Corresponding author.

- Case report design
- Data collection
- Data interpretation
- Writing the paper

## Registration of research studies

N/A.

## Guarantor

The corresponding author is the guarantor of this manuscript.

## Consent

Written informed consent was obtained from the patient for publication of this case report and accompanying images.

## Provenance and peer review

Not commissioned, externally peer reviewed.

## Declaration of competing interest

No conflicts of interest were identified in the writing of this case report.
